# Donor-Derived Genotype 4 Hepatitis E Virus Infection, Hong Kong, China, 2018

**DOI:** 10.3201/eid2503.181563

**Published:** 2019-03

**Authors:** Siddharth Sridhar, Vincent C.C. Cheng, Shuk-Ching Wong, Cyril C.Y. Yip, Shusheng Wu, Anthony W.I. Lo, Kit-Hang Leung, Winger W.N. Mak, Jianpiao Cai, Xin Li, Jasper F.W. Chan, Susanna K.P. Lau, Patrick C.Y. Woo, Wai-Ming Lai, Tze-Hoi Kwan, Timmy W.K. Au, Chung-Mau Lo, Sally C.Y. Wong, Kwok-Yung Yuen

**Affiliations:** The University of Hong Kong, Hong Kong, China (S. Sridhar, V.C.C. Cheng, S.-C. Wong, C.C.Y. Yip, S. Wu, K.-H. Leung, W.W.N. Mak, J. Cai, X. Li, J.F.W. Chan, S.K.P. Lau, P.C.Y. Woo, C.-M. Lo, S.C.Y. Wong, K.-Y. Yuen);; Queen Mary Hospital, Hong Kong (A.W.I. Lo, T.W.K. Au);; Princess Margaret Hospital, Hong Kong (W.-M. Lai); Tuen Mun Hospital, Hong Kong (T.-H. Kwan)

**Keywords:** hepatitis E virus, hepatitis E, transplantation, immune system, chronic hepatitis, hepatitis, ribavirin, Hong Kong, China, viruses

## Abstract

Hepatitis E virus (HEV) genotype 4 (HEV-4) is an emerging cause of acute hepatitis in China. Less is known about the clinical characteristics and natural history of HEV-4 than HEV genotype 3 infections in immunocompromised patients. We report transmission of HEV-4 from a deceased organ donor to 5 transplant recipients. The donor had been viremic but HEV IgM and IgG seronegative, and liver function test results were within reference ranges. After a mean of 52 days after transplantation, hepatitis developed in all 5 recipients; in the liver graft recipient, disease was severe and with progressive portal hypertension. Despite reduced immunosuppression, all HEV-4 infections progressed to persistent hepatitis. Four patients received ribavirin and showed evidence of response after 2 months. This study highlights the role of organ donation in HEV transmission, provides additional data on the natural history of HEV-4 infection, and points out differences between genotype 3 and 4 infections in immunocompromised patients.

Hepatitis E virus (HEV; genus *Orthohepevirus*) is a major cause of hepatitis globally (*1*). In immunocompetent persons, it typically causes self-limited acute hepatitis. In immunocompromised persons, such as transplant recipients, HEV infection can persist, causing chronic hepatitis and cirrhosis (*2*). HEV-A, the main HEV virus of relevance to human health, is classified into 8 genotypes, of which 5 can infect humans (*3*). Persistent hepatitis E is most commonly reported for patients infected with HEV genotype 3 (HEV-3), the prevalent genotype infecting humans in Europe and the Americas (*4*–*8*). In many parts of China, HEV genotype 4 (HEV-4) has rapidly emerged as the most common genotype causing acute hepatitis (*9*,*10*). We recently demonstrated that, similar to HEV-3, HEV-4 can cause persistent infections in transplant recipients (*11*). However, data on the natural history and ribavirin responsiveness of HEV-4 infections in immunocompromised patients are limited.

HEV-3 and HEV-4 are predominantly transmitted by food, specifically undercooked pork products. Transmission through blood product transfusion has also been well documented (*12*,*13*), prompting many countries to consider screening blood products for HEV (*14*). Less frequently reported is HEV transmission via transplanted organs (*15*,*16*).

In August 2018, chronic HEV-4 infection developed in 2 transplant recipients who had received organs from a common donor. In response, we conducted an investigation to 1) identify other infected recipients from the same donor, 2) confirm that the infection was indeed donor derived, 3) investigate the natural history of HEV-4 infection, and 4) study outcomes of infected patients who received ribavirin. 

## Materials and Methods

### Study Setting, Patients, and Samples

In Hong Kong, Queen Mary Hospital is the liver and heart–lung transplantation center, Princess Margaret Hospital is a kidney transplant center, and Tuen Mun Hospital offers specialist follow-up services for kidney transplant recipients. HEV diagnostic testing for this study was performed at the University of Hong Kong Department of Microbiology, based at Queen Mary Hospital. We retrieved patient identifiers for the organ donor and recipients from the organ donor registry and retrieved clinical details for donor and recipients from the electronic patient record. Archived serum samples from the donor and all recipients were retrieved and subjected to HEV quantitative real-time reverse transcription PCR (qRT-PCR) and serologic testing. Ethics approval for this study was obtained from the institutional review board of the University of Hong Kong/Hospital Authority Hong Kong West Cluster.

### HEV Diagnostic Testing, Sequencing, and Phylogenetic Analysis

For serologic testing, we used HEV IgM and HEV IgG ELISA kits (Wantai, http://www.ystwt.cn/HEV.html). In addition, we tested donor and recipient serum samples by using IgG and IgM Western blots as previously described (*17*). HEV qRT-PCR was performed as previously described (*18*). Protocols for the Western blot, HEV qRT-PCR, sequencing, and phylogenetic analysis are described in the Appendix.

### Liver Histology and Immunohistochemistry

Liver tissue sections from the organ donor and liver graft recipient were available for histologic analysis with hematoxylin and eosin staining. We performed immunohistochemical staining with monoclonal antibody against HEV open reading frame 2 (kindly provided by N.S. Xia, Xiamen University, Xiamen, China) on tissue sections, as previously described (*19*).

### Case Definitions

Patients were defined as having hepatitis E if HEV qRT-PCR detected HEV RNA in their plasma. Patients were defined as having probable donor-derived hepatitis E if they had received organ transplant from the viremic donor and subsequently hepatitis E developed. To prove that the infections were definitely donor derived, we phylogenetically compared recipient HEV sequences with the original donor sequence. Persistent HEV infection was defined as detection of HEV RNA in plasma for >3 months (*20*). If archived samples were unavailable, persistent hepatitis for 3 months before the first positive HEV RNA result was also defined as persistent HEV infection.

## Results

### The Outbreak

**Figure 1:**
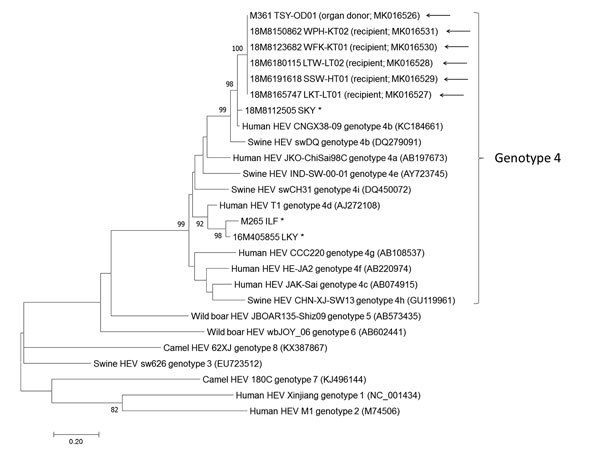
Phylogenetic analyses of the partial RNA-dependent RNA polymerase region of HEV strains involved in study of donor-derived genotype 4 HEV infection, Hong Kong, China, 2018, and other HEV genotypes. The bootstrap analysis was performed with 1,000 replicates. Bootstrap values <70% are not shown. The analysis included 382 nt positions. GenBank accession numbers are shown in parentheses. Asterisks (*) indicate locally identified cases of HEV infection; arrows (←) indicate HEV outbreak cases from this study. Scale bar indicates estimated number of substitutions per site. HEV, hepatitis E virus.

**Figure 2:**
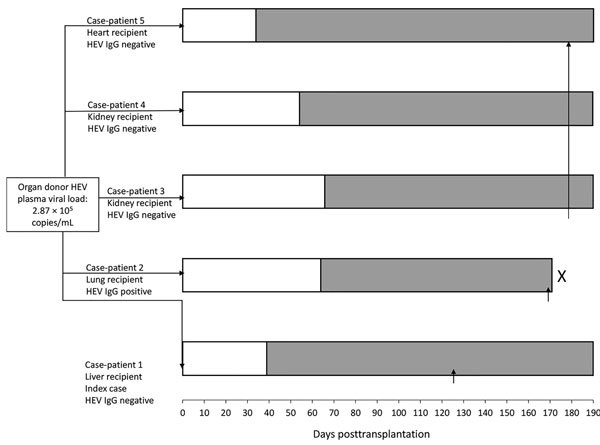
Timeline of outbreak in study of donor-derived genotype 4 HEV infection, Hong Kong, China, 2018, showing baseline HEV IgG status of each organ recipient. White bars indicate incubation period during which liver function test results were within reference range. Gray bars indicate timeline of alanine aminotransferase derangement after transplantation. X indicates patient death. Vertical arrows (↑) indicate time of hepatitis E diagnosis. HEV, hepatitis E virus.

On posttransplantation day 127, detection of HEV RNA in plasma from a liver transplant recipient at Queen Mary Hospital led to a diagnosis of hepatitis E, at the time attributed to autochthonous foodborne acquisition. On posttransplantation day 162, HEV RNA was detected in plasma from a lung transplant recipient at Queen Mary Hospital. Both patients had undergone transplantation at Queen Mary Hospital on February 28, 2018, and had received organs from the same deceased donor. Archived donor serum was tested by HEV qRT-PCR and found to be positive. Because both kidneys and the heart had also been harvested from this donor, samples were collected from the 3 corresponding recipients, and HEV RNA was detected in the plasma of all 3. Phylogenetic analysis confirmed that HEV partial RNA-dependent RNA polymerase sequences from the donor and 5 organ recipients were identical and belonged to genotype 4b (Figure 1). All 5 recipients fulfilled criteria for persistent HEV infection according to the case definition used in this study. Except for the lung transplant recipient, who died before hepatitis E infection could be confirmed, all patients were counseled about the diagnosis and prescribed ribavirin. When possible, their immunosuppression was reduced. For each patient, plasma and serum samples archived before and after transplantation were retrieved for HEV qRT-PCR and serology testing. The timeline of the outbreak is depicted in Figure 2, and individual case details are summarized in Table 1.

**Table 1:** Characteristics of 5 patients who received organs from the same donor and had donor-derived genotype 4 hepatitis E virus infection, Hong Kong, China, 2018*

Case-patient	1	2	3	4	5
Organ transplanted	Liver	Lung	Kidney	Kidney	Heart
Age, y/sex	66/M	59/M	6/M	54/F	48/F
Incubation period, d†	40	65	67	54	34
Signs/symptoms	Ascites	None	None	None	None
Peak ALT level, U/L	1,385	138	490	186	130
Lymphocytes at hepatitis onset, × 10^9^ cells/L	0.43	0.59	1.4	0.37	1.12
Pretransplantation HEV IgG‡	–	+	–	–	–
Pretransplantation HEV-IgG§	–	–	–	–	–
Posttransplantation HEV-IgM/HEV-IgG¶	+/+	+/+	−/−	+/−	−/−
Posttransplantation HEV-IgM/HEV-IgG§	+/+	+/+	−/−	+/+	−/−

*All patients had received tacrolimus, mycophenolate, and prednisolone before onset of hepatitis E. ALT, alanine aminotransferase; HEV, hepatitis E virus.

†Time between transplantation and onset of abnormal ALT level.

‡HEV IgG ELISA (http://www.ystwt.cn/HEV.html).

§Western blot.

¶HEV IgM/HEV IgG ELISA (http://www.ystwt.cn/HEV.html).

### Organ Donor

The organ donor was a previously healthy 29-year-old woman admitted to Princess Margaret Hospital for acute subarachnoid hemorrhage that led to brainstem death. Alanine aminotransferase (ALT) level was 18 U/L (reference range 8–58 U/L), aspartate aminotransferase (AST) 22 U/L (reference range 15–38 U/L), and bilirubin 16 μmol/L (reference range 4–23 μmol/L). On hospitalization day 4 (February 28, 2018), her heart, liver, lungs, and kidneys were harvested and transplanted to 5 recipients. After hepatitis E in the organ recipients was recognized, HEV qRT-PCR was performed on archived donor serum collected on the day of organ harvest; viral load was 2.87 × 10^5^ copies/mL. This serum sample was negative for HEV IgM and IgG by both ELISAs and the Western blot (Appendix Figure 1). HEV RNA was detected in liver graft tissue; viral load was 2.88 × 10^2^ copies/reaction. Histology of liver graft tissue showed minimal inflammation at portal tracts, and immunohistochemical staining that used HEV monoclonal antibodies did not show positive signals (Appendix Figure 2, panels A, B).

### Case-Patient 1 (Liver Graft Recipient)

**Figure 3:**
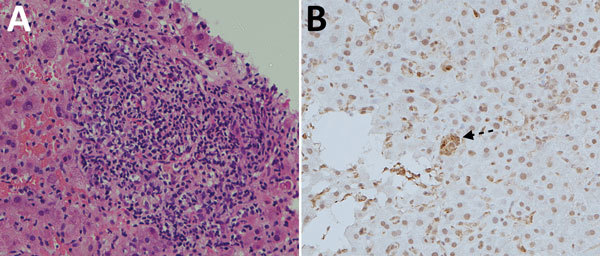
Histology of tissue from liver graft of hepatitis E virus case-patient 1, a 66-year-old man, on posttransplantation day 122. A) Hematoxylin and eosin staining showing moderate (grade 2) inflammation. B) Immunohistochemical staining (using hepatitis E virus monoclonal antibody); arrow indicates small groups of hepatocytes with positive cytoplasmic signals. Original magnification ×200.

**Figure 4:**
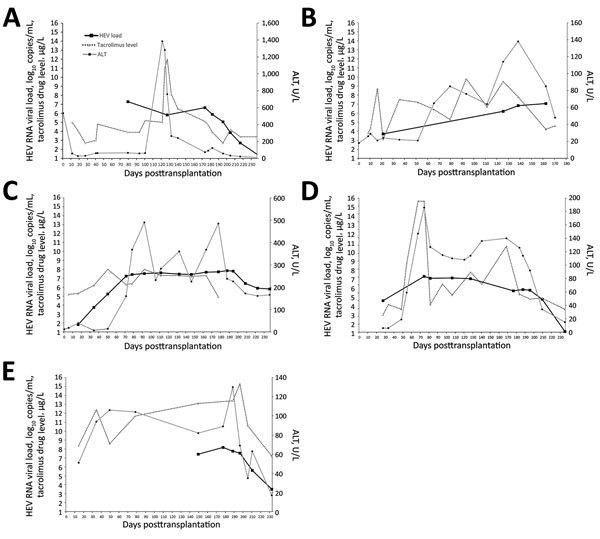
Kinetics of liver function test (ALT) results, tacrolimus levels, and plasma HEV RNA load with relation to ribavirin therapy. A) Case-patient 1; B) case-patient 2; C) case-patient 3; D) case-patient 4; E) case-patient 5. Date for case-patients 1, 3, 4, and 5 were updated up to week 8 of ribavirin treatment. Horizontal black bars indicate when patient began taking oral ribavirin. ALT, alanine aminotransferase; HEV, hepatitis E virus.

The index case-patient was a 66-year-old man with a history of liver cirrhosis caused by hepatitis C virus infection that had been cured with direct-acting antiviral medications. During his liver transplantation, he received basiliximab and hydrocortisone for intraoperative immunosuppression. Postoperatively, immunosuppression was maintained with tacrolimus, mycophenolate, and prednisolone. After transplantation, his liver function test results initially normalized, but on posttransplantation day 40, ALT rose to 62 U/L. Liver function test results abruptly worsened, reaching a nadir on posttransplantation day 123 (bilirubin 85 μmol/L, ALT 1,478 U/L, and AST 561 U/L). Prothrombin time was elevated to 14.2 seconds. Liver histology on posttransplantation day 122 showed moderate inflammation at portal tracts (Figure 3, panel A). Acute organ rejection was treated by increasing immunosuppression. Hepatitis E was diagnosed after HEV RNA was detected in EDTA-treated blood collected on posttransplantation day 127. Immunohistochemical staining of the posttransplantation day 122 liver biopsy sample, with monoclonal antibodies against HEV, showed granular cytoplasmic staining of groups of hepatocytes (Figure 3, panel B). HEV RNA load in this biopsy sample was 1.21 × 10^7^ copies/reaction. Because the patient’s liver function test results were spontaneously improving (Figure 4, panel A), immunosuppression was reduced and HEV RNA loads monitored. However, the patient was repeatedly admitted for ascites, and HEV RNA was persistently detected in plasma with mildly elevated ALT. In view of persistent HEV infection and portal hypertension, oral ribavirin (200 mg/400 mg on alternate days, dose adjusted for renal impairment) was started on posttransplantation day 177; viremia cleared within 2 months (Figure 4, panel A).

### Case-Patient 2 (Lung Transplant Recipient)

A 59-year-old man with a history of bronchiectasis and chronic obstructive pulmonary disease underwent sequential bilateral lung transplantation. The procedure was complicated by bleeding requiring exploratory thoracotomy for hemostasis. For postoperative immunosuppression, the patient was administered tacrolimus, mycophenolate, and prednisolone. The patient required prolonged hospitalization for ventilator-associated pneumonia. Starting at posttransplantation day 65, he had low-grade hepatitis. ALT was elevated to 65 U/L, AST to 45 U/L, while bilirubin remained within normal limits. Over the next 3 months, ALT elevations persisted (Figure 4, panel B). On posttransplantation day 161, the patient experienced generalized tonic–clonic convulsion and vesicular rash over the groin. Disseminated herpes zoster was confirmed by detection of varicella zoster virus DNA in cerebrospinal fluid, plasma, and swab samples from the vesicular rash. Despite intravenous acyclovir, the patient’s condition progressively deteriorated to refractory shock, coagulopathy, and then death on posttransplantation day 171. Postmortem qRT-PCR testing of a plasma sample sent on posttransplantation day 162 confirmed a diagnosis of hepatitis E. An archived sample from posttransplantation day 21 that had been positive for HEV RNA confirmed persistent HEV infection (Figure 4, panel B). IgG in baseline pretransplant serum was detectable by ELISA, but this finding could not be confirmed by Western blot (Appendix Figure 1).

### Case-Patient 3 (Kidney Transplant Recipient)

A 6-year-old boy with focal segmental glomerulosclerosis underwent kidney transplantation at Princess Margaret Hospital. Immunosuppression was induced with methylprednisolone and basiliximab and maintained with oral tacrolimus, mycophenolate, and prednisolone. Mycophenolate was switched to azathioprine because of BK virus reactivation. Starting at posttransplantation day 67, persistent ALT level abnormalities were noted. Because hepatitis E had been diagnosed in 2 other recipients of organs from the same donor, HEV qRT-PCR testing of plasma collected on posttransplantation day 177 was performed, and results confirmed the diagnosis of persistent HEV infection. Administration of tacrolimus and azathioprine was stopped, and immunosuppression was continued with cyclosporin A, everolimus, and prednisolone. On posttransplantation day 180, oral ribavirin (100 mg 2×/d) was started. Liver function test results improved; HEV viral load underwent a 1-log reduction after the patient had received ribavirin for 1 month (Figure 4, panel C). However, during the second month of treatment, viral load and liver function test results plateaued.

### Case-Patient 4 (Kidney Transplant Recipient)

A 54-year-old woman with chronic glomerulonephritis underwent kidney transplantation at Princess Margaret Hospital. Preoperatively, she received antithymocyte globulin and methylprednisolone. Postoperative immunosuppression included tacrolimus, mycophenolate, and prednisolone. During follow-up at Tuen Mun Hospital on posttransplantation day 54, abnormal parenchymal enzymes were first noted, coinciding with an increasing trend in tacrolimus levels detected in May (Figure 4, panel D); in addition, ALT was elevated to 59 U/L, ALP was 442 U/L, and bilirubin levels were within reference range at 8 μmol/L. The result of HEV qRT-PCR of plasma collected on posttransplantation day 176 was positive. Tacrolimus dosage was reduced, and oral ribavirin (400 mg 1×/day) was started. Within 2 months of treatment, ALT levels normalized and viremia cleared (Figure 4, panel D).

### Case-Patient 5 (Heart Transplant Recipient)

A 49-year-old woman received a heart transplant for severe myocarditis at Queen Mary Hospital. Intraoperatively, she received 500 mg intravenous methylprednisolone; postoperatively, as an outpatient, she received immunosuppression with tacrolimus, mycophenolate, and prednisolone. On posttransplantation day 34, abnormal liver function test results were first noted; ALT was 123 U/L, AST 94 U/L, and ALP 123 U/L, but bilirubin levels were within reference range. Subsequent blood testing showed ongoing low-grade hepatitis (Figure 4, panel E). Because other transplant-transmitted hepatitis E infections from the same donor had been recognized, HEV qRT-PCR was performed on posttransplantation day 177; plasma contained an HEV RNA load of 1.51 × 10^8^ copies/mL. Because the hepatitis had persisted for >3 months, on posttransplantation day 181, oral ribavirin (200 mg/d, adjusted for renal function) was started, and all immunosuppressant dosages were reduced. At the end of the second month of ribavirin treatment, ALT and HEV RNA loads were improving.

## Discussion

This hepatitis E outbreak affected 5 transplant recipients who had received organs from a donor with HEV viremia. Two previous studies have described HEV transmission from organ donors to recipients (*15*,*16*). In both of those studies, liver function test results were abnormal for donors at the time of organ donation; 1 donor had subclinical hepatic HEV carriage (without serum HEV IgM or RNA positivity), and the other had acute hepatitis E (with serum HEV IgM and RNA positivity) (*15*,*16*). The donor in our study was a young woman whose liver function test results were within reference ranges and whose premortem serum was negative for HEV IgM but positive for HEV RNA; these values are compatible with organ donation during the window period of hepatitis E infection. However, subclinical long-term HEV carriage without seroconversion, a recently described entity in immunocompetent blood donors (*21*,*22*), cannot be excluded. Adoptive transfer of functional hepatitis B immunity via transplantation has been reported (*23*,*24*). The lack of measurable HEV humoral antibodies in the donor’s serum may have facilitated HEV transmission in the absence of adoptive transfer of anti-HEV immune responses.

The risk for HEV transmission during transplantation has been recognized in the United Kingdom and Spain (*25*,*26*); guidelines from both countries recommend organ donor HEV screening. This recommendation is further supported by a recent study that reported that 1 (0.95%) of every 105 liver grafts is contaminated with HEV (*27*). The screening method of choice is nucleic acid amplification testing (NAAT), which is more sensitive than serologic testing (*13*,*28*). Undetectable HEV RNA in donor serum would indicate low risk for HEV transmission, but the experience of Schlosser et al. suggests that liver grafts may harbor infectious HEV RNA even in the absence of systemic markers of infection (*15*). The most sensitive screening method may be HEV NAAT of graft tissue samples obtained at the time of transplantation. Although NAAT results may not be available before transplantation, the detection of HEV RNA in graft tissues could be used to guide posttransplant management of recipients. However, in resource-limited settings, the decision to implement universal organ donor HEV screening would pose substantial difficulties. Furthermore, even if the organ donor is HEV negative, recipients remain vulnerable to HEV infection through dietary and blood product transfusion routes. Ultimately, the decision to screen organ donors will depend on trends in HEV incidence in the general population. Screening organ donors in areas of low HEV prevalence may not add much value to transplant safety.

In our study, for all 5 organ recipients, hepatitis developed within a mean of 52 days after transplantation. The kidney, heart, and lung recipients showed no symptoms of hepatitis, but the liver recipient had severe hepatitis. This discrepancy is probably the consequence of an inflammatory response to high HEV antigenic load in the liver graft. Although the hepatitis in the liver graft recipient was temporarily suppressed by an increased dosage of immunosuppressants, graft dysfunction progressed to portal hypertension and refractory ascites, eventually requiring treatment with ribavirin.

HEV IgG is considered to provide cross-genotypic protection against HEV infection (*29*,*30*). In this outbreak, both ELISA and the Western blot results indicated that 4 of 5 patients were HEV IgG negative before transplantation, indicating absence of protective immunity from previous exposure. This finding is consistent with our previous findings of high HEV susceptibility among transplant recipients in Hong Kong (*31*).

The HEV strain in the outbreak reported here belonged to genotype 4b. Although some studies found that HEV-4 acute infection is more severe than HEV-3 infection (*32*,*33*), this finding has not been corroborated by a recent systematic review (*34*). Few data on the clinical characteristics of HEV-4 infection in immunocompromised patients are available (*11*,*35*–*38*). Of the 7 reported cases of HEV-4 infection in immunocompromised patients, 6 cases progressed to persistent infection; in 3 cases, infection did not respond to ribavirin or relapsed despite ribavirin (Table 2). All 5 patients in our study experienced persistent HEV-4 infection. Combined with the findings of our previous study (*11*), we note that persistent HEV-4 infection developed in 8 (89%) of 9 transplant recipients with hepatitis E in Hong Kong. In 4 patients from this study (case-patients 1–4) and 2 from our previous study (*11*), immunosuppression was reduced without any effect on HEV-4 viral load. This experience contrasts with findings of a previous seminal study in which HEV-3 infection cleared spontaneously even without reduction of immunosuppression in 34% of HEV-3 infected transplant recipients and cleared after reduction of immunosuppression in another 21% (*39*). The patients in our study were either taking tacrolimus or mTOR (mammalian target of rapamycin) inhibitors, which are risk factors for viral persistence (*39*,*40*). However, the effect of HEV genotype on persistence of infection requires further exploration.

**Table 2:** Summary of studies describing HEV-4 infections in immunocompromised patients

Reference	Patient age, y/sex	Underlying immunosuppressive condition/treatment	Peak ALT, U/L	Progression to persistent HEV infection	Ribavirin treatment/ response	Patient outcome
(*37*)	4/M	Acute lymphoblastic leukemia	585	Spontaneous clearance, relapsed 20 mo later	No	Spontaneous clearance of relapse; resolution of hepatitis
(*36*)	68/M	Liver transplantation/tacrolimus	149	Yes	Yes/yes	Required retransplantation because of accelerated liver fibrosis; patient died of hemorrhage
(*35*)	47/F	Liver transplantation/tacrolimus	Not reported	Yes	Yes/no	Progressive cholestasis; patient died of sepsis
(*11*)	52/M	Renal transplantation/prednisolone, cyclosporin A, and everolimus or sirolimus	230	Yes	Yes/no	Ribavirin resistance; progressive cirrhosis
	55/M		456	Yes	Yes/yes	Resolution of hepatitis
	65/M		470	Yes	Yes/yes	Resolution of hepatitis
(*38*)	36/M	Renal transplantation/ tacrolimus	300	Yes	Yes/yes	Relapse after ribavirin withheld; progressive hepatitis

*ALT, alanine aminotransferase; HEV, hepatitis E virus.

All 4 surviving patients received ribavirin, which has been shown to be effective for treating persistent HEV-3 infections (*41*). At the time this article was written (≈2 months after patients started taking ribavirin), ALT levels had improved for all 4 patients, and viremia had cleared for 2 patients. Reduction of HEV viral load was slower for case-patient 3 and plateaued at the end of the second month of treatment. Further follow-up of this patient is needed to confirm whether this is ribavirin refractory disease, which we have previously described for HEV-4 infections (*11*).

Our study has several limitations. We were unable to ascertain the exact route of infection in the donor because of the long duration (≈6 months) between organ donation and recognition of the outbreak. Because archived blood samples obtained from recipients shortly after transplantation were not available, we were unable to comprehensively examine the viral load dynamics of HEV infection. The variable performance of HEV serologic assays and lack of a standard serologic test is a limitation of our study, which we tried to minimize by using 2 independent assay formats. Although some research findings suggest that the analytical sensitivity and specificity of the Wantai HEV IgG and IgM ELISAs are good, the work of Cattoir et al. shows that discordant results with other commercial assays are not uncommon (*42*,*43*). In our study, we found 100% concordance in HEV IgM detection between both formats, but the Western blot was able to detect HEV IgG in 1 more case-patient than the Wantai ELISA. Furthermore, the Wantai ELISA result indicated that case-patient 2 had HEV IgG in pretransplant serum, but this finding was not confirmed by Western blot. 

We recognize that definitive conclusions on HEV-4 infections cannot be based on limited case series. To delineate intergenotypic differences in clinical characteristics, natural history, and ribavirin responsiveness, prospective studies of HEV-4 infection in cohorts of immunocompromised patients are needed. 

Appendix Additional methods and results for study of donor-derived genotype 4 hepatitis E virus infection, Hong Kong, China, 2018.
