# Correction: Vol. 25, No. 3

**DOI:** 10.3201/eid2506.C22506

**Published:** 2019-06

**Keywords:** errata, erratum, correction

Figures 2 and 4 were incomplete in Donor-Derived Genotype 4 Hepatitis E Virus Infection, Hong Kong, China, 2018 (S. Sridhar et al.). The corrected figures are reproduced here, and the article has been corrected online (https://wwwnc.cdc.gov/eid/article/25/3/18-1563_article).

