# Supplementary material for: Donor-Derived Genotype 4 Hepatitis E Virus Infection, Hong Kong, China, 2018
Source: Emerg Infect Dis. 2019 Mar;25(3):425–33. doi: 10.3201/eid2503.181563 (PMC6390757; doi:10.3201/eid2503.181563)
Supplement: Appendix — Additional methods and results for study of donor-derived genotype 4 hepatitis E virus infection, Hong Kong, China, 2018. [file 18-1563-Techapp-s1.pdf]

Article DOI: https://doi.org/10.3201/eid2503.181563

Donor-Derived Genotype 4 Hepatitis E Virus Infection, Hong Kong, China, 2018

Appendix

1.1 Hepatitis E virus quantitative reverse transcription real-time PCR (HEV qRT-PCR)

Viral total nucleic acid (TNA) was extracted from plasma/serum samples using NucliSENS easyMAG extraction system (bioMérieux, Marcy-l'Étoile, France). TNA was eluted in 25 µl and was used as template for reverse transcription polymerase chain reaction (RT-PCR). RNA was extracted from liver tissue using TRIzol reagent (ThermoFisher, Waltham, Massachusetts, United States) and eluted into a final volume of 40 µL.

Samples were subjected to HEV qRT-PCR using primers and conditions probe targeting the HEV ORF3 gene (*1*). Real-time one-step RT-PCR assay was performed using QuantiNova Probe RT-PCR Kit (Qiagen, Germany) in LightCycler 480 Real-Time PCR System (Roche Diagnostics, Basel, Switzerland). Each 20µl-reaction mix contained 1x QuantiNova Probe RT-PCR Master Mix, 1x QN Probe RT-Mix, 0.8 µM forward and reverse primers, 0.2 µM probe and 5 µl template TNA. Reactions were incubated at 45°C for 10 min and 95°C for 5 min, followed by 50 cycles at 95°C for 5 s and 55°C for 30 s.

1.2 HEV sequencing

Reverse transcription was performed using the SuperScript IV kit (Invitrogen, Carlsbad, California, USA). HEV partial RNA-dependent RNA polymerase (RdRp) gene was amplified and sequenced by using forward primer 5′-ATAACATTCTTYCAGAARGAYTG-3′ and reverse primer 5′-CTCACCRGARTGYTTCTTCCA-3′. All PCR products were gel-purified using the QIAquick gel extraction kit (Qiagen, Hilden, Germany). Both strands of the PCR products were sequenced with 3500 Genetic Analyzer (Applied Biosystems, Foster City, California, USA) using the PCR primers.

1.3 Phylogenetic analysis

Phylogenetic tree was constructed using the maximum-likelihood method by MEGA 7.0 program (*2*). A Tamura-Nei model with gamma distribution and invariant sites (G+I) was used.

1.4 Western blot

A 239 aa peptide fragment of HEV-4 ORF2 was cloned and expressed as previously described (*3*). Purified protein was separated electrophoretically in a 12% gel and transferred to a nitrocellulose membrane. Western blot was performed in a Mini-PROTEAN II Multiscreen Apparatus (Bio-Rad, Hercules, CA, USA). The membrane was incubated with sera from the organ donor, all five recipient's baseline and post-transplant sera (at a dilution of 1:500) as well as known positive and negative controls for 1 h at 37°C. After washing, the membrane was incubated with horseradish peroxidase (HRP; Sigma-Aldrich, St. Louis, MO, USA) conjugated goat anti-human (IgG or IgM) and goat anti-mouse antibodies for 30 min at 37°C, and developed by incubation with Advansta ECL WesternBright Quantum Detection Kit (Advansta, Menlo Park, CA, USA).

Appendix References

1. Jothikumar N, Cromeans TL, Robertson BH, Meng XJ, Hill VR. A broadly reactive one-step real-time RT-PCR assay for rapid and sensitive detection of hepatitis E virus. J Virol Methods. 2006;131:65–71. PubMed http://dx.doi.org/10.1016/j.jviromet.2005.07.004

2. Kumar S, Stecher G, Tamura K. MEGA7: Molecular Evolutionary Genetics Analysis Version 7.0 for Bigger Datasets. Mol Biol Evol. 2016;33:1870–4. PubMed http://dx.doi.org/10.1093/molbev/msw054

3. Sridhar S, Yip CCY, Wu S, Cai J, Zhang AJ, Leung KH, et al. Rat hepatitis E virus as cause of persistent hepatitis after liver transplant. Emerg Infect Dis. 2018;24:2241–50. PubMed http://dx.doi.org/10.3201/eid2412.180937

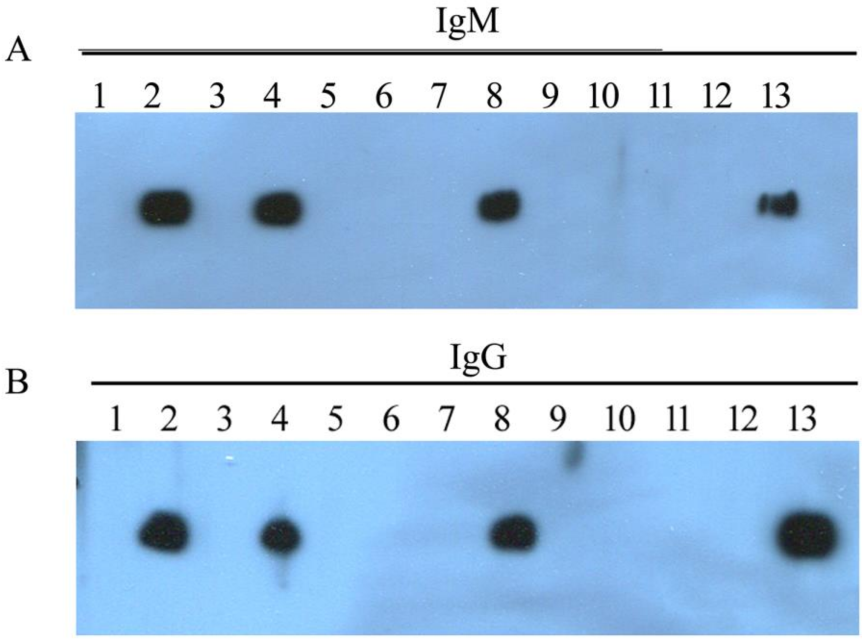

Appendix Figure 1. Result of IgM (A) and IgG (B) Western blot using HEV-4 purified recombinant protein. Lane 1: lung transplant recipient pre-transplant serum, lane 2: lung transplant recipient post-transplant serum, lane 3: kidney transplant recipient (case-patient 4) pre-transplant serum, lane 4: kidney transplant recipient (case-patient 4) post-transplant serum, lane 5: kidney transplant recipient (case-patient 3) pre-transplant serum, lane 6: kidney transplant recipient (case-patient 3) post-transplant serum, lane 7: liver transplant recipient pre-transplant serum, lane 8: liver transplant recipient post-transplant serum, lane 9: heart transplant recipient pre-transplant serum, lane 10: heart transplant recipient post-transplant serum, lane 11: organ donor serum, lane 12: negative control human serum, lane 13: positive control human serum.

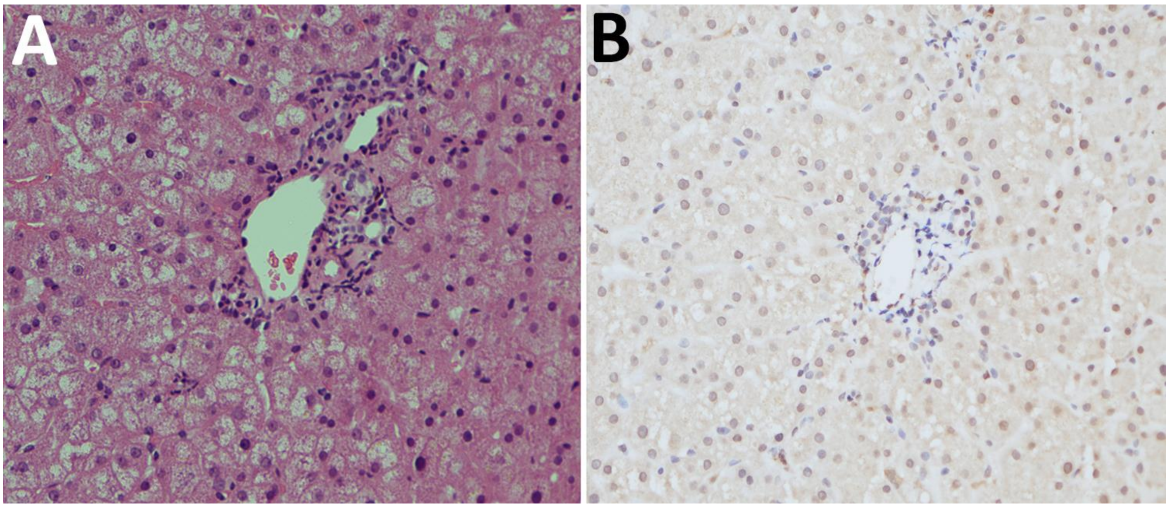

Appendix Figure 2. H&E stained section of the liver graft on day of transplantation showed minimal inflammation at the portal tracts (A); immunohistochemical staining of liver graft on day of transplantation using anti-HEV monoclonal antibody revealed no definite signals (B).
